# Adding a fatigue item to the EQ-5D-5L improves its psychometric performance in the general population

**DOI:** 10.1186/s41687-021-00406-x

**Published:** 2022-01-04

**Authors:** Inge Spronk, Suzanne Polinder, Gouke J. Bonsel, M. F. Janssen, Juanita A. Haagsma

**Affiliations:** 1 Department of Public Health, Erasmus MC, University Medical Center Rotterdam, P.O. Box 2040, 3000 CA Rotterdam, The Netherlands; 2 Association of Dutch Burn Centres, Maasstad Hospital, Rotterdam, the Netherlands; 3 EuroQol Group Executive Office, Rotterdam, The Netherlands; 4 Section Medical Psychology and Psychotherapy, Department of Psychiatry, Erasmus MC, Rotterdam, The Netherlands

**Keywords:** EQ-5D, Health-related quality of life, Fatigue, Bolt-on, General population

## Abstract

**Background:**

Fatigue is a common and often disturbing sequela of serious chronic health conditions. In the widely applied HRQL instrument, the EQ-5D, this aspect is not included directly, for its assumed lack of additional information. We investigated the validity of this assumption by determining the gain—if any—of an additional fatigue item to the EQ-5D-5L in a general population sample.

**Methods:**

A Dutch general population sample (including diseased people) completed a web-based survey including the EQ-5D-5L and the Rivermead Post-Concussion Symptoms Questionnaire (RPQ). The RPQ fatigue item was used to create the EQ-5D-5L + Fatigue. We head-to-head compared the psychometric performance contrasting the EQ-5D-5L and EQ-5D-5L + Fatigue: distribution (e.g. ceiling), informativity cf. Shannon's indices, convergent validity, domain dependency, and explanatory power. Results were compared between subgroups with and without ≥ 1 chronic health condition.

**Results:**

The study population consisted of 3027 persons of whom 52% had a chronic health condition. The mean EQ-5D-5L utility score was 0.83 and 48% experienced some degree of fatigue. Adding the fatigue item to the EQ-5D-5L decreased the ceiling effect, increased absolute informativity (Hʹ = 6.44 vs. Hʹ = 4.90) and relative informativity (Jʹ = 0.46 vs. Jʹ = 0.42). The extra fatigue item slightly increased convergent validity (Spearman’s rank correlation coefficient = − 0.61 vs. − 0.62). Domain dependency analysis showed that all EQ-5D-5L domains are dominant over the fatigue item. Explanatory power of the EQ-5D-5L + Fatigue was higher compared to the EQ-5D-5L (R^2^ = 0.42 vs. 0.39). The gain is substantially larger in the subgroup with chronic health conditions.

**Conclusions:**

Adding a fatigue item to the EQ-5D-5L improved all psychometric performance criteria of the enriched instrument in the general population. Effects are substantially larger in the subgroup with chronic health conditions, indicating that adding a fatigue item to the EQ-5D-5L is especially relevant in evaluating the HRQL of diseased people.

## Background

Self-reported health-related quality of life (HRQL) questionnaires are increasingly used to evaluate the impact of a health condition, the effectiveness of treatments and interventions, and the level of quality of care [1–3]. HRQL instruments aim to capture the patient's perception of his/her physical, psychological and social wellbeing [4]. HRQL instruments can be generic (i.e. applicable to any disease or condition) or disease/condition-specific. Disease-specific instruments focus on one specific condition and may include treatment-defined levels of ill-health, i.e. thresholds defining start or stop of a particular treatment [5]. Generic instruments allow for comparison between different diseases, between subgroups of the general population, and often they allow for use within multi-faceted diseases [5]. All measures are a compromise between feasibility (i.e. the ease of an instrument in its intended context of use, given constraints such as time or money), on the one hand, and validity (i.e. the degree to which an instrument measures the constructs it purports to measure) and reliability on the other hand [6].

The EQ-5D is a widely used generic HRQL instrument both in economic and clinical applications, that has been validated for a wide range of diseases and is available in many languages [7, 8]. An advantage is its conciseness and low burden to complete this instrument [9, 10]. The EQ-5D consists of five domains: mobility, self-care, usual activities, pain/discomfort, and anxiety/depression. The EQ-5D also includes a visual analogue scale (EQ VAS) [11]. The five domains can be scored on either a three (EQ-5D-3L) or more refined five level (EQ-5D-5L) ordinal scale [11, 12]. Based on these five scores an index with preference interpretation can be calculated by applying weights which have been separately derived. To cover lack of comprehensiveness, the EQ-5D enables the use of ‘bolt-on’ items [13, 14]. A ‘bolt-on’ is a item (like fatigue or cognition) on a specific health problem or dysfunction that is not included in the original instrument [13].

This paper addresses the construct validity and sensitivity of EQ-5D for fatigue problems. Together with sensory problems and cognitive problems, fatigue is often mentioned as potential deficit in the domain structure of the EQ-5D [13, 15–17]. During the inception of the EQ-5D instrument an 'energy/tiredness' was considered as the positively defined inverse of 'fatigue'. It was not included in the final version of the instrument as it failed to show additional effect in small-sized analyses [18–20].

In active disease and quite some chronic health conditions, the relevance of fatigue to the patient is beyond doubt [21–24]. Focused recent studies suggest that in generic measures (like EQ-5D-5L) the fatigue/energy/tiredness domain could be considered as relevant addition [15, 16, 25–28]. A recent study listing 17 specific health-related features not addressed by the EQ-5D, put fatigue forward as the feature most needed [15].

The general population also reports some degree of fatigue, up to 50% [29–31]. While this limits the domain to be used as disease classifier, it enables research into the coverage of fatigue by other EQ-5D domains using HRQL data from a general population sample. The primary aim of this study is to what extent adding a fatigue item would add unique information to the generic EQ-5D-5L. The secondary aim is to detail the results for people with and without a chronic disease separately.

## Methods

### Participants

A web-based survey was distributed to members of the Dutch general population aged 18–75 years by Survey Sampling International during the period June 29th till July 31st 2017 [32]. The sample was selected to be representative of the Dutch population with respect to sex, age, and education. All participants completed an informed consent form before completing the survey. The study was part of the large CENTER-TBI study (EC grant 602150). Ethical approval was provided by the Leids Universitair Centrum—Commissie Medische Ethiek (P14.222/NV/nv).

### Measures

As background information, the survey collected data on age, sex, highest achieved level of education, household income level, and self-reported chronic health conditions. Several health measures were included. The first was a generic measure: the EQ-5D-5L, which assesses a patient’s HRQL and health status today. The default response format is five ordered response options (1–5; no problems, slight problems, moderate problems, severe problems, and extreme problems) [12]. Based on the five domain scores, a utility score (through weighting) was calculated at the individual level, using the Dutch value set [33]. The utility score is anchored at 0 (referring to a state as bad as being dead) and 1 (full health) [9]. This study also used an equally weighted sum of all domain scores, range 5–25, the 'level sum score'. For descriptive analysis the scores of the five domains can be combined to create a health profile; profile ‘11111’ represents the best possible health state given the classification, while profile ‘55555’ represents the worst health state. This composed score is different from the level sum score as the specific score on each domain is indicated, whereas in the level sum score, the domain scores are summed up into one overall score without knowing what domain contributed to a higher score. In addition, the EQ-5D includes a visual analogue scale (EQ VAS) [34] that assesses the respondent’s health on a scale from 0 (worst imaginable health) to 100 (best imaginable health) [9].

The second instrument was the Rivermead Post-Concussion Symptoms Questionnaire (RPQ) [35]. This questionnaire lists 16 different symptoms and complaints (including 'fatigue'), with five ordinal response options. The single fatigue item of the RPQ runs: ‘Do you (i.e., over the last 24 h) suffer from fatigue?’. Response options included: 0 (not experienced at all), 1 (no more of a problem), 2 (a mild problem), 3 (a moderate problem) and 4 (a severe problem). To resemble the 5L format of the EQ-5D, the 0–4 score was recoded into a 1–5 score.

### Data analyses

Descriptive statistics were used to assess characteristics and outcomes of the EQ-5D-5L and the RPQ fatigue item. EQ-5D-5L and RPQ fatigue outcomes were compared between subgroups of respondents with and without ≥ 1 chronic health condition. Mann–Whitney U tests were used for continuous variables and chi-square tests for categorical variables.

The RPQ fatigue item was used to investigate the added value of a fatigue item for the EQ-5D-5L. The EQ-5D-5L + Fatigue was created by combining the EQ-5D-5L with the recoded RPQ fatigue item. First, we studied distributional effects of the EQ-5D-5L and EQ-5D-5L + Fatigue by analyzing the number of unique profiles and the ceiling of the EQ-5D-5L vs. the EQ-5D-5L + Fatigue. The ceiling was calculated by the comparison of full health profiles, ‘11111’ for the EQ-5D-5L and ‘111111’ for the EQ-5D-5L + Fatigue. A higher proportion of full health profiles indicates a higher degree of ceiling. Also, the relation between fatigue and the EQ VAS was investigated by studying the mean EQ VAS for each of the five levels of fatigue. The same relation was studied for the five most frequently observed EQ-5D health states.

Then, the informativity of the EQ-5D-5L and EQ-5D-5L + Fatigue was assessed by calculation of the Shannon index (Hʹ) and the Shannon Evenness index (Jʹ) [36–38]. These Shannon indices give information on the ability of the EQ-5D-5L(+ Fatigue) to reflect and quantify diversity in a population [39]. The Shannon index (Hʹ) was determined by: ${\text{H}}^{\prime } = - \sum\nolimits_{{\text{i = 1}}}^{{\text{c}}} {{\text{pi}}^{{2}} {\text{log p}}_{{\text{i}}} }$. With pi being the proportion of respondents with one specific health profile (e.g., 11111), and C being the total number of theoretically possible health profiles (i.e., 3125 for the EQ-5D-5L, and 15,625 for the EQ-5D-5L + Fatigue). If a profile is not observed its contribution is 0 (zero) to this summation. A higher Shannon index (Hʹ) indicates that more information is captured by the EQ-5D-5L or EQ-5D-5L + Fatigue. The Shannon Evenness index (Jʹ) was calculated as: Jʹ = Hʹ/Hʹmax, with Hʹmax is 2logC. The Shannon Evenness index (Jʹ) reflects whether the extra domain is used to discriminate more health profiles as efficient as the standard set—in that case J' remains unchanged while H' will increase [40]. Both Hʹ and Jʹ need to be taken into account for a full interpretation of informativity when comparing classifications [40]. We computed H' and J' for the EQ-5D-5L and the EQ-5D-5L + Fatigue, separately.

Convergent validity was assessed by analyzing the Spearman’s rank correlation between the EQ-5D-5L (level sum score) and the EQ VAS, and the EQ-5D-5L + Fatigue (level sum score) and the EQ VAS. Cohen’s criteria were applied to evaluate the strength of association: correlations were strong if r ≥ 0.50, moderate if r ≥ 0.30–0.49, and weak if r ≥ 0.10–0.29 [41]. For determination of domain dependency (redundancy) we studied mutual relations between the domains. A domain A is defined to dominate another domain B, if the likelihood that a poor level in A coincides with a good level B is smaller than the probability of the reverse situation, corrected for chance. It is a simple indicator of directionality of relations between domains in a given dataset. These probabilities were presented in domain-to-domain cross tables of the EQ-5D-5L domains and the fatigue item. Profiles with a severe or extreme problem in one domain (L4: severe problem or L5: extreme problem) and no problem (L1: no problem) in another domain were analyzed [42]. For example, profiles with fatigue level 4/5 and mobility level 1 were compared to profiles with mobility level 4/5 and fatigue level 1. The ratio of the chance adjusted frequencies provides information on the dominance of a domain: if the result of the equation is equal to 1 the domains are independent; if the result of the equation is < 1 fatigue dominates; if the result of the equation is > 1 fatigue is subordinate or dependent [33].

Lastly, the added explanatory power of an extra fatigue item was tested through regression analyses, with the EQ VAS score as outcome. Predictive performance was compared of the EQ-5D-5L domains with and without the extra fatigue item in univariate and multivariable analyses. The levels ‘slight problems’ (L2), ‘moderate problems’ (L3), ‘severe problems’ (L4) and ‘extreme problems’ (L5) were used to predict the EQ VAS. The ‘no problems’ level (L1) was used as the reference category. Then, multivariable analyses were performed with combinations of the five EQ-5D domains and the fatigue item in the model. The combinations consisted of the original EQ-5D-5L domains, the EQ-5D-5L + Fatigue dimensions, and all combinations of five out of the six domains. All analyses were done for the whole sample as well as for the two subgroups with (diseased group) and without ≥ 1 chronic health condition (healthy group). With the significance level of all analyses was set at *p* < 0.05. All analyses were done in IBM SPSS Statistics 25.

### Hypotheses

- Health-related quality of life is better and the proportion and severity of fatigue lower in respondents without a chronic health condition compared to those with ≥ 1 chronic health conditions.
- The ceiling (proportion persons in the best profile) in EQ-5D-5L + Fatigue is lower compared to EQ-5D-5L.
- Adding a fatigue item increases absolute informativity (Hʹ) of the EQ-5D-5L in terms of Shannon's indices; while relative informativity (Jʹ) remains comparable.
- Adding a fatigue item increases convergent validity of the EQ-5D-5L, using the EQ VAS as reference.
- Adding a fatigue item increases explanatory power no less or more than any other domain, using the EQ VAS as reference.
- The added value of fatigue is larger in the subgroup with ≥ 1 chronic health condition compared to the subgroup without a chronic health condition.

## Results

### Respondents

In total, 3027 people returned the questionnaire. The mean age was 44.7 years old (SD 15.3) and half of the participants was male (Table 1). Most had a middle level of education (46.9%), and the majority was employed (54.0%). About half of the participants (52.0%) had one or more chronic health condition (31.2% one condition; 19.9% two or more conditions).

**Table 1:** Characteristics of study population

Characteristic	Total sample (n = 3027)
*Sex*: Male	1520 (50.2%)
*Age* (M, SD)	44.7 (15.3)
*Age categories*	
18–24 years	365 (12.1%)
25–39 years	814 (26.9%)
40–59 years	1231 (40.7%)
60–75 years	617 (20.4%)
*Level of education*	
Low	811 (26.8%)
Middle	1420 (46.9%)
High	796 (26.3%)
Work status*	
Employed	1635 (54.0%)
Unemployed	316 (10.4%)
Looking after others	125 (4.1%)
Student	209 (6.9%)
Retired	386 (12.8%)
Unable to work	356 (11.8%)
*Number of chronic health conditions*	
No chronic health condition	1453 (48.0%)
1 chronic health condition	971 (32.1%)
2 chronic health conditions	368 (12.2%)
3 chronic health conditions	149 (4.9%)
≥ 4 chronic health conditions	86 (2.8%)

*Work status was categorized as employed (employee and self-employed), unemployed (consisting out of work for more than and less than 1 year), looking after others (e.g. a carer or parent), a student, retired and unable to work

### EQ-5D-5L and fatigue outcomes

The mean EQ-5D-5L utility score was 0.83 (SD 0.21) and the mean EQ VAS was 76.3 (SD 18.1) (Table 2). Most problems were reported for the pain/discomfort domain; 51.4% of the respondents reported at least slight problems. Least problems were reported for self-care; 8.7% reported any problem. A total of 47.9% of the respondents reported at least mild fatigue problems. The mean EQ-5D-5L utility score and EQ VAS score were significantly higher ('better') in healthy respondents without a chronic health condition compared to the diseased group (*p* < 0.001). A significantly higher proportion of diseased respondents reported fatigue compared to healthy respondents (*p* < 0.001). The diseased group showed more problems for all domains (*p* < 0.001), the contrast was largest in the pain/discomfort domain (73.3% vs. 27.8%).

**Table 2:** EQ-5D-5L and fatigue outcomes

	Total sample (n = 3027)	Without a chronic health condition (n = 1453)	With ≥ 1 chronic health condition (n = 1574)	*p*-value
*EQ-5D-5L outcomes*				
Mobility (% with problems)	819 (27.1%)	123 (8.5%)	696 (44.2%)	< 0.001
Self-care (% with problems)	264 (8.7%)	28 (1.9%)	236 (15.0%)	< 0.001
Usual activities (% with problems)	917 (30.3%)	116 (8.0%)	801 (50.9%)	< 0.001
Pain/discomfort (% with problems)	1557 (51.4%)	404 (27.8%)	1153 (73.3%)	< 0.001
Anxiety/depression (% with problems)	998 (33.0%)	267 (18.4%)	731 (46.4%)	< 0.001
Utility score (M, SD)	0.83 (0.21)	0.93 (0.11)	0.73 (0.23)	< 0.001
*EQ VAS*	76.3 (18.1)	83.8 (13.7)	69.5 (19.0)	< 0.001
*RPQ Fatigue*				< 0.001
Not experienced at all	1195 (39.5%)	810 (55.7%)	385 (24.5%)	
No more of a problem	381 (12.6%)	191 (13.1%)	190 (12.1%)	
Mild fatigue	708 (23.4%)	300 (20.6%)	408 (25.9%)	
Moderate fatigue	478 (15.8%)	123 (8.5%)	355 (22.6%)	
Severe fatigue	265 (8.8%)	29 (2.0%)	236 (15.0%)	

### Distributional effects—ceiling, unique profiles

In total, 1116 of the 3027 respondents (37%) reported full health with the EQ-5D-5L, while 744 (25%) reported full health with the fatigue item added. The 372 respondents that had a full health status based on the EQ-5D-5L, but not based on the EQ-5D-5L + Fatigue were on average 40.4 years old (SD 14.8) and slightly more than half of them were females (53%). Most had a middle education (48%), and the minority had a chronic health condition (27%). The 25% full health group with the fatigue item added had a somewhat higher mean age (44.5 years old; SD 15.6) and lower presence of chronic health conditions (17% instead of 27%).

The number of observed unique health profiles (among all respondents) was 368 out of 3125 (12%) for the EQ-5D-5L, and 636 out of 15,625 (4%) for the EQ-5D-5L + Fatigue. In the healthy group (n = 1453), 90 out of 3125 (3%) possible EQ-5D-5L health profiles, and 156 out of 15,625 (1%) possible EQ-5D-5L + Fatigue health profiles were observed. In the diseased group (n = 1574) respondent 342 out of 3125 (11%) possible EQ-5D-5L health profiles, and 581 out of 15,625 (4%) possible EQ-5D-5L + Fatigue health profiles were observed.

Table 3 shows the mean EQ VAS score for each level of fatigue and any EQ-5D outcome, as well as for the five most common EQ-5D profiles. Mean EQ VAS score decreased when the severity of fatigue problems increased. Mean EQ VAS was 84.3 when no fatigue was present, 75.9 when patients experienced mild fatigue problems, and 54.7 when patients experienced severe fatigue problems. The same pattern was more or less present in the selected EQ-5D profiles, except for the profile ‘11111’, where the mean EQ VAS was similar in patients with severe fatigue problems (mean EQ VAS = 84.9) compared to patients with mild fatigue (mean EQ VAS = 83.5) and higher when compared to patients with moderate fatigue problems (mean EQ VAS = 78.4), although these differences were not significantly different.

**Table 3:** EQ VAS score for each of the five recoded fatigue items, and for the five most common EQ-5D-5L profiles

EQ-5D profile	Fatigue = 1		Fatigue = 2		Fatigue = 3		Fatigue = 4		Fatigue = 5	
	n	Mean EQ VAS (95% CI)	n	Mean EQ VAS (95% CI)	n	Mean EQ VAS (95% CI)	n	Mean EQ VAS (95% CI)	n	Mean EQ VAS (95% CI)
All	1195	84.3 (83.5–85.1)	381	78.0 (76.5–79.6)	708	75.9 (74.8–77.0)	478	67.8 (66.3–69.4)	265	54.7 (52.1–57.2)
*Subgroups based on their EQ-5D profile*										
11111	744	87.5 (86.5–88.4)	132	85.2 (83.5–86.8)	174	83.5 (81.8–85.2)	50	78.4 (74.0–82.8)	16	84.9 (78.3–91.6)
11121	155	85.1 (83.3–86.9)	52	83.1 (80.8–85.4)	78	81.1 (78.9–83.4)	37	81.9 (78.7–85.2)	7	78.0 (67.7–88.4)
11112	46	80.6 (76.1–85.1)	26	75.7 (68.0–83.4)	55	79.8 (76.1–83.6)	27	79.0 (75.8–82.2)	0	
11122	28	77.4 (70.8–84.0)	13	75.4 (61.8–89.0)	40	78.1 (75.0–81.2)	28	73.9 (69.3–78.4)	10	72.7 (59.9–85.5)
21221	28	81.0 (75.0–87.0)	19	78.6 (75.1–82.2)	18	73.6 (64.4–82.8)	14	77.5 (70.7–84.3)	3	78.7 (58.4–90.0)

### Informativity of the EQ-5D-5L with and without an extra fatigue item

The Shannon Index (Hʹ) was 4.90 for the EQ-5D-5L and 6.44 for the EQ-5D-5L + Fatigue. The Shannon Evenness index (Jʹ) of the EQ-5D-5L was lower than that of the EQ-5D-5L + Fatigue (0.422 vs. 0.462). In healthy respondents, Hʹ was 2.58 and Jʹ was 0.222 for the EQ-5D-5L, while Hʹ was 4.05 and Jʹ was 0.291 for the EQ-5D-5L + Fatigue. In the disease group Hʹ was 6.41 and Jʹ was 0.552 for the EQ-5D-5L and Hʹ was 7.90 and Jʹ was 0.567 for the EQ-5D-5L + Fatigue.

### Convergent validity

The Spearman’s rank correlation coefficient between the EQ-5D-5L (level sum score) and the EQ VAS was − 0.606 (strong), and − 0.621 (strong) between the EQ-5D-5L + Fatigue (level sum score) and the EQ VAS. In the diseased group these coefficients were − 0.331 (moderate), and − 0.369 (moderate) respectively. Correlations almost doubled in the diseased group: − 0.614 (strong) and − 0.637 (strong) respectively.

### Domain dependency

Table 4 shows the degree to which domains dominated each other. Severe/extreme problems on fatigue and no problems on other items were relatively common (5.5–20.1%), especially for no problems on self-care (20.1%) and mobility (12.4%). In contrast, no problems on fatigue and severe/extreme problems on other items were very uncommon (0.2–0.7%). Relative frequency analysis showed that the ratio of ‘fatigue level 4/5—other domain level 1’ and ‘other domains level 1—fatigue level 4/5’ was > 1 for all five domains, indicating that the EQ-5D-5L domains are dominant over fatigue.

**Table 4:** Combinations of extreme values of the EQ-5D-5L + Fatigue (no versus severe/extreme problems), in absolute numbers and expressed as the percentage of the total number of profiles

Extreme problems (L4/L5)	No problems (L1)					
	Mobility	Self-care	Usual activities	Pain/discomfort	Anxiety/depression	Fatigue
Mobility		41 (1.4%)	11 (0.4%)	9 (0.3%)	46 (1.5%)	17 (0.6%)
Self-care	6 (0.2%)		8 (0.3%)	6 (0.2%)	8 (0.3%)	6 (0.2%)
Usual activities	16 (0.5%)	40 (1.3%)		10 (0.3%)	26 (0.9%)	7 (0.2%)
Pain/discomfort	35 (1.2%)	124 (4.1%)	26 (0.9%)		84 (2.8%)	22 (0.7%)
Anxiety/depression	56 (1.9%)	80 (2.6%)	22 (0.7%)	23 (0.8%)		9 (0.3%)
Fatigue	375 (12.4%)	608 (20.1%)	276 (9.1%)	165 (5.5%)	296 (9.8%)	

### Explanatory power of the EQ-5D-5L with and without a fatigue item

Univariate analyses showed substantial impact of fatigue levels 4 (moderate fatigue) and 5 (severe fatigue) (Appendix 1). Table 5 shows the multivariable regression analyses (all respondents, and healthy and diseased respondents separately). In all respondents, 38.7% of the variance of the EQ VAS is explained by the five EQ-5D-5L domains. The addition of the fatigue item (41.6%) provided extra explanatory power. In respondents without a chronic health condition, the explained variance increased from 15.6 to 17.5% due to the addition of the fatigue item, whereas it increased from 35.5 to 39.6% in the disease group.

**Table 5:** Explanatory power of multivariable models for the EQ VAS that include EQ-5D-5L domains with and without an extra fatigue item added

Selection of EQ-5D-5L + Fatigue items	R^2^	df	F-value	*p*-value
*Total sample*				
MO + SC + UA + PD + AD	0.387	20	94.8	< 0.001
SC + UA + PD + AD + FA	0.410	20	104.3	< 0.001
MO + UA + PD + AD + FA	0.413	20	105.7	< 0.001
MO + SC + PD + AD + FA	0.406	20	102.8	< 0.001
MO + SC + UA + AD + FA	0.390	20	96.2	< 0.001
MO + SC + UA + PD + FA	0.397	20	98.8	< 0.001
MO + SC + UA + PD + AD + FA	0.416	24	89.0	< 0.001
*Without chronic health condition*				
MO + SC + UA + PD + AD	0.156	19	14.0	< 0.001
SC + UA + PD + AD + FA	0.167	18	16.0	< 0.001
MO + UA + PD + AD + FA	0.175	20	15.2	< 0.001
MO + SC + PD + AD + FA	0.161	19	14.4	< 0.001
MO + SC + UA + AD + FA	0.147	19	14.2	< 0.001
MO + SC + UA + PD + FA	0.149	19	13.2	< 0.001
MO + SC + UA + PD + AD + FA	0.175	23	13.2	< 0.001
*With chronic health condition*				
MO + SC + UA + PD + AD	0.355	20	42.8	< 0.001
SC + UA + PD + AD + FA	0.382	19	50.7	< 0.001
MO + UA + PD + AD + FA	0.392	20	50.0	< 0.001
MO + SC + PD + AD + FA	0.385	20	48.7	< 0.001
MO + SC + UA + AD + FA	0.368	20	45.2	< 0.001
MO + SC + UA + PD + FA	0.378	20	47.1	< 0.001
MO + SC + UA + PD + AD + FA	0.396	24	42.3	< 0.001

MO, mobility; SC, self-care; UA, Usual activities; PD, Pain/discomfort; AD, Anxiety/depression; FA, Fatigue

## Discussion

This study demonstrated, in a large sample of the general Dutch population, that an additional fatigue item provides additional information to the standard EQ-5D. As hypothesized, the ceiling effect decreased by adding the extra fatigue item and more information is captured. The extended EQ-5D-5L + Fatigue version was better able to differentiate between respondents compared to the EQ-5D-5L, especially between those with a chronic health condition. Severe/extreme fatigue problems were shown to be associated with a steep decrease in EQ VAS scores and convergent validity showed that the EQ-5D-5L + Fatigue is more strongly related to the EQ VAS compared to the EQ-5D-5L. Compared to the EQ-5D-5L domains, the extra fatigue item added most explanatory power, especially in the subgroup of respondents with a chronic health condition.

Our finding that fatigue provides additional information and is a candidate item to be added to the EQ-5D is in line with less elaborate previous studies that suggested to add a fatigue (or related) item to the EQ-5D [15–17, 25, 26, 28, 43]. Despite the use of different approaches, all showed that the EQ-5D would benefit from an additional fatigue or related item. These studies entailed related constructs: energy/sleep [43], fatigue [15], energy/fatigue [17], tiredness [16, 25, 26]. Only one study tested the psychometric gains of adding a fatigue item to the EQ-5D, demonstrating a decreased ceiling effect [17]. They also studied the explanatory power of adding the energy/fatigue item and showed that it added 5% to the EQ-5D-3L in a sample of the Swiss general population. When considering fatigue to add as a bolt-on item to the EQ-5D, it should be clarified what construct (i.e. energy, tiredness, fatigue) is best. An earlier study showed that energy was less important to add compared to hearing and cognition, though it is unclear whether this also applies when the construct fatigue is used [44]. Also, as the wording and terminology may have impact on the outcomes, this should be carefully investigated as well. If fatigue is added as a bolt-on item to the EQ-5D, an important next step is modelling the tariff of the EQ-5D to include the bolt-on item while not substantially altering the weighting system [45].

As fatigue is an important sequela of many chronic health conditions, we studied the potential gain of adding a fatigue item to the EQ-5D separately for the subgroups of respondents with and without a chronic health condition. As hypothesized, especially in the subgroup of respondents with a chronic health condition the extra fatigue item captures additional information and improves the coverage of HRQL. For all psychometric aspects studied, the gain of the extra fatigue item was substantially higher for the subgroup of respondents with a chronic health condition, particularly the informativity, convergent validity and the explanatory power. The explanatory power of the EQ-5D-5L increased with 0.9% in the subgroup without a chronic health condition versus 4.1% in the subgroup of respondents with a chronic health condition when the fatigue item was added.

This study included several strengths and limitations. One of the strengths was the large sample size that was representative of the Dutch population for age, sex and educational level. Another strength was the prevalence of having a chronic health condition, which was comparable to the Dutch population (52% vs. 58%) [46]. Other strengths included the ability to divide the sample in large subgroups with and without a chronic health condition facilitating the comparison of these subgroups, and the high prevalence and wide range of fatigue scores allowing to study the gain of adding a fatigue item to the EQ-5D-5L. A limitation was, however, the application of the RPQ fatigue item to assess fatigue. The RPQ has been developed for the assessment of post-concussion symptoms. It is not specifically developed for the assessment of fatigue in the general population. However, an earlier study showed the ability of the RPQ fatigue item to assess fatigue in a general population [32]. Another limitation was the slightly different phrasing of the timeframe (your health today vs. fatigue in the past 24 h). For future studies it is recommended to add fatigue as a sixth domain to the EQ-5D to study fatigue as a bolt-on item and overcome these issues when assessing the additional value of fatigue. It would also be interesting to study the added value of a fatigue bolt-on in populations with a specific condition for which fatigue is a major consequence [21–24]. Also, a limitation was the web-based form of the survey. By using this method, we were not aware of the characteristics of non-respondents, and we were consequently not able to study whether respondents differed from non-respondents.

## Conclusions

The present study showed that adding a fatigue item to the EQ-5D-5L improves the discriminatory power, informativity, convergent validity and explanatory power of the EQ-5D-5L. Effects are substantially larger in the subgroup of respondents with chronic health conditions, indicating that adding a fatigue item to the EQ-5D-5L is especially relevant in evaluating the HRQL of diseased people.

## Data Availability

The datasets generated and/or analysed during the current study are not publicly available due to privacy/ethical restrictions but are available from the corresponding author on reasonable request.

## Appendix 1

See Table 6.

**Table 6:** Univariate analyses of the EQ VAS explained by the EQ-5D-5L and extra fatigue item

	R-Square	Unstandardized B (95% CI)	*p*-value
Mobility level 2	0.044	− 10.4 (− 12.1 to − 8.7)	< 0.001
Mobility level 3	0.071	− 18.2 (− 20.5 to − 15.8)	< 0.001
Mobility level 4	0.052	− 24.9 (− 28.6 to − 21.1)	< 0.001
Mobility level 5	0.001	− 8.4 (− 16.6 to − 0.3)	0.043
Self-care level 2	0.066	− 18.6 (− 21.5 to − 15.8)	< 0.001
Self-care level 3	0.032	− 20.6 (− 24.6 to − 16.6)	< 0.001
Self-care level 4	0.013	− 31.0 (− 40.4 to − 21.5)	< 0.001
Self-care level 5	0.000	− 5.6 (− 14.8 to 3.6)	0.235
Usual Activities level 2	0.044	− 9.9 (− 11.6 to − 8.3)	< 0.001
Usual Activities level 3	0.121	− 21.7 (− 23.8 to − 19.6)	< 0.001
Usual Activities level 4	0.044	− 24.7 (− 28.8 to − 20.6)	< 0.001
Usual Activities level 5	0.015	− 27.0 (− 34.9 to − 19.1)	< 0.001
Pain/discomfort level 2	0.000	− 0.3 (− 1.1 to 1.7)	0.690
Pain/discomfort level 3	0.071	− 14.0 (− 15.8 to − 12.2)	< 0.001
Pain/discomfort level 4	0.129	− 26.9 (− 29.3 to − 24.4)	< 0.001
Pain/discomfort level 5	0.026	− 29.2 (− 35.7 to − 22.8)	< 0.001
Anxiety/depression level 2	0.027	− 7.5 (− 9.0 to − 5.9)	< 0.001
Anxiety/depression level 3	0.051	− 14.2 (− 16.4 to − 12.0)	< 0.001
Anxiety/depression level 4	0.043	− 23.6 (− 27.6 to − 19.7)	< 0.001
Anxiety/depression level 5	0.005	− 12.7 (− 19.0 to − 6.4)	< 0.001
Fatigue level 2	0.001	− 1.9 (− 0.03 to − 3.9)	0.052
Fatigue level 3	0.000	− 0.6 (− 2.2 to 0.9)	0.413
Fatigue level 4	0.041	− 10.1 (− 11.9 to − 8.4)	< 0.001
Fatigue level 5	0.137	− 23.7 (− 25.9 to − 21.6)	< 0.001

## Abbreviations

EQ VAS: EQ-5D visual analogue scale
HRQL: Health-related quality of life
L1-5: Level 1–5
RPQ: Rivermead Post-Concussion Symptoms Questionnaire
SD: Standard deviation
